# Supplementary figures and images for: Acute repeated cage exchange stress modifies urinary stress and plasma metabolic profiles in male mice
Source: PLoS One. 2023 Oct 10;18(10):e0292649. doi: 10.1371/journal.pone.0292649 (PMC10564260; doi:10.1371/journal.pone.0292649)

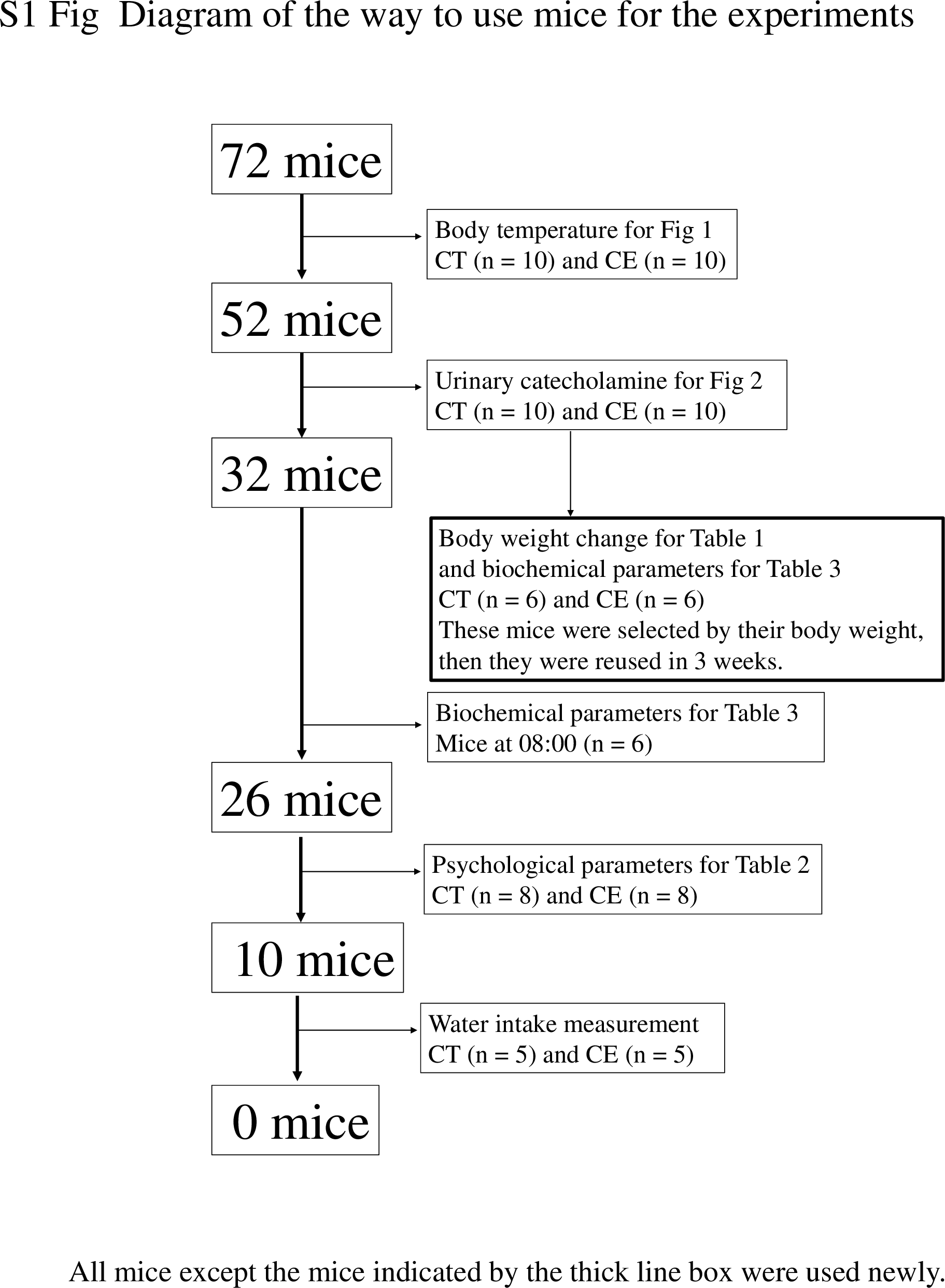

Supplement: S1 Fig — (TIF) [file pone.0292649.s001.tif]
