# Supplementary material for: Acute repeated cage exchange stress modifies urinary stress and plasma metabolic profiles in male mice
Source: PLoS One. 2023 Oct 10;18(10):e0292649. doi: 10.1371/journal.pone.0292649 (PMC10564260; doi:10.1371/journal.pone.0292649)
Supplement: Attachment — Submitted filename: Letter to the editor for the revised manuscript.docx [file pone.0292649.s007.docx]

Yukiori Goto, Ph.D.

Academic Editor

PLOS ONE

Dear Prof. Goto,

We are pleased to learn that our manuscript (PONE-D-23-13098, Acute repeated cage exchange stress modifies urinary stress and plasma metabolic profiles in male mice) can be considered for publication in your journal.

Journal Requirements

**1. Please review your reference list to ensure that it is complete and correct. If you have cited papers that have been retracted, please include the rationale for doing so in the manuscript text, or remove these references and replace them with relevant current references. Any changes to the reference list should be mentioned in the rebuttal letter that accompanies your revised manuscript. If you need to cite a retracted article, indicate the article’s retracted status in the References list and also include a citation and full reference for the retraction notice.**

We have checked the reference list. We have confirmed that it has no papers that have been retracted. We have formatted the list as ordered by the journal.

Additionally, we noticed the error of the version of the EZR for statistical analysis. We changed the number in the revised text.

**Reviewer #3: Thank you for your revisions on the manuscript.**

**A couple of minor notes:**

**Line 84 – Please add in a sentence to describe a few aspects as to how brain function is altered in response to body temperature changes.**

We added the words “for fight-or-flight”.

**Lines 98-102 – This last section is difficult to read/understand; I would recommend separating into at least two sentences.**

We separated the sentence.

In this paper, we report the changes in nutrients, especially amino acids, which have limited information regarding their responses to stress and are consumed to meet energy demand. We used a stress model in which the animals can still move freely, namely, repeated cage exchange (CE), mimicking exposure to a novel environment.

**Lines 138-142 – It is still difficult to understand which animals were used for each measure. It would be good to insert a diagram here starting from 72 mice, using arrows to separate to different groups of animals that were used for the different experiments/measures. In addition, groups of animals should be represented as n values. For example: Body temperature: Group A includes n=10 CT and n=10 CE (Figure 1); Urinary catecholamines and creatine: Group B includes n=10 CT and n=10 CE (Figure 2); Group C, etc… It would also be appropriate to highlight where a group of animals were used over multiple measures.**

We made a diagram showing the way to use mice in the respective experiments. Then, we put it as a supporting figure (S1 Fig), which is indicated in Line 143.

**Lines 171-172 and 174: This should read ‘anxiety-like behaviour’ rather than ‘anxiety-like conditions’. Please make any other changes to this wording throughout the manuscript.**

We changed the word or the word like that to ‘anxiety-like behavior’ in any places.
